# Circulating visfatin concentrations in patients with chronic obstructive pulmonary disease: systematic review and meta-analysis

**DOI:** 10.3389/fmed.2024.1432025

**Published:** 2025-01-07

**Authors:** Nahid Aboutaleb, Alireza Moradi, Hamzeh Mirshekari Jahangiri, Mohammad Reza Aslani

**Affiliations:** ^1^Physiology Research Center, Iran University of Medical Sciences, Tehran, Iran; ^2^Lung Diseases Research Center, Ardabil University of Medical Sciences, Ardabil, Iran

**Keywords:** visfatin, meta-analysis, COPD, IL-6, BMI

## Abstract

**Objective:**

The current study was designed with the aim of conducting a systematic review and meta-analysis to determine the circulating levels of visfatin in patients with chronic obstructive pulmonary disease (COPD) compared to healthy individuals.

**Methods:**

Until March 2024, we searched the Web of Science, PubMed/Medline, and Scopus databases. The analysis included case–control studies assessing the association between circulating visfatin and COPD. The random effects model was utilized to analyse the results with the help of Standard Mean of Differences (SMD) and 95% confidence interval (CI). The heterogeneity of the data was assessed using Cochrane Q and I^2^ values.

**Results:**

Seven studies were eligible to be included in the meta-analysis, with the COPD and healthy (control) groups having 265 and 244 subjects, respectively. The pooled results showed that although the circulating concentration of visfatin was lower in patients with COPD, no significant difference was observed (SMD: −0.48 mg/L; 95% CI: −1.67 to 0.70; *p* = 0.43). Subgroup analysis revealed that visfatin levels were significantly reduced in FEV1 less than 50% (*p* < 0.001) and in GOLD grade I-II (*p* < 0.05). Visfatin was shown to be significantly associated with IL-6 (*p* < 0.001) and TNF-α (*p* < 0.01) in the correlation meta-analysis. Meta-regression analysis revealed a significant correlation between the pooled SMD visfatin and pooled SMD age (*p* < 0.01), BMI (*p* < 0.001), FEV1 (*p* < 0.001), and IL-6 (*p* < 0.001).

**Conclusion:**

The findings showed an insignificant decline in visfatin level among COPD patients, but additional research is necessary due to the heterogeneity in study results.

**Systematic review registration:**

PROSPERO (CRD42023450851), https://www.crd.york.ac.uk/prospero/display_record.php?ID=CRD42023450851.

## 1 Introduction

Airflow limitation and chronic respiratory symptoms are distinctive of chronic obstructive pulmonary disease (COPD). COPD patients can suffer from restricted airflow due to the destruction of lung parenchyma or airway disease (1–3). COPD, a complex disorder, is caused by a range of factors including genetic predisposition, cigarette smoke exposure, dietary habits, occupational hazards, physical activity, lifestyle, and air pollution both indoors and outdoors (4, 5). The pathophysiology of COPD is affected by a variety of factors, such as neutrophilic airway inflammation, an imbalance of proteases and antiproteases, oxidative stress, endoplasmic reticulum stress, tissue hypoxia, lung hyperinflation, and apoptosis (6–8). Elevated levels of cytokines and inflammatory markers such as interleukin-6 (IL-6), tumour necrosis factor-alpha (TNF-α), IL-8, and C-reactive protein (CRP) have been observed in the airways of COPD patients during exacerbation (9, 10). In addition, results from recent investigations have revealed the key contribution of adipokines [such as leptin, adiponectin, visfatin, adipolin, and fatty acid binding protein 4 (FABP4)] released from adipose tissue in the pathogenesis of chronic lung diseases (11–13).

Visfatin is an adipokine formerly known as B-cell colony-enhancing factor (PBEF) and nicotinamide phosphoribosyltransferase (NAMPT) (14). The majority of studies have posited visfatin as pro-inflammatory factor, due to the significant positive correlation between visfatin levels and inflammatory markers (15, 16). Moreover, inflammatory markers are able to prompt the secretion of visfatin, and visfatin itself can also evoke the secretion of inflammatory components (14). Despite numerous studies exploring the connection between visfatin and COPD, the outcomes have been profoundly conflicting. Consequently, this systematic review and meta-analysis study was aimed at assessing the circulating levels of visfatin among COPD patients and determining its potential association with inflammatory factors.

## 2 Methods

The present systematic review and meta-analysis were performed according to the Preferred Reporting Items for Systematic Reviews and Meta-Analyses (PRISMA) strategies (17). In addition, the study protocol was registered in PROSPERO (CRD42023450851).

### 2.1 Search strategy

From inception to March 2024, the electronic databases of PubMed / Medline, the Web of Sciences, the Scopus, as well as manual search in article references and Google Scholar were searched for studies examining the circulating level of visfain in COPD patients. The mesh and non-mesh terms utilized in the searching were summarized in Supplementary Table 1. The language requirement for articles was English, but no time limits were imposed. Moreover, a manual search was done of all applicable article reference lists to recognize possibly applicable studies.

### 2.2 Eligibility criteria

The criterion for the current meta-analysis study was designed based on population, intervention, comparison, and outcome (PICO) (Table 1). No correspondence was entered into with the authors.

**Table 1:** The population, intervention, comparison, outcome, study design (PICOS) criteria.

Criteria (1)	Selection criteria
Population	Adults (aged ≥18 years)
Intervention	COPD patients
Comparison	Healthy subjects
Outcome	Circulating visfatin concentrations
Study design	Case–Control

COPD, Chronic obstructive pulmonary disease.

To be included, the criteria were: (1) observational studies, (2) COPD patients in accordance with the American or European Pulmonary Association guidelines, (3) with a sample size of more than 10 subjects, and (4) healthy participants with no evidence of disease or infection. The exclusion criteria, if mentioned in the articles, were: (1) patients with a history of a lung disease outside of COPD; (2) patients receiving a supportive nutritional treatment; (3) patients receiving specific drugs; (4) comorbidity that could affect circulating visfatin levels.

### 2.3 Data extraction

Human studies in which visfatin serum/plasma levels in patients with COPD compared to healthy subjects were included in the meta-analysis. Two researchers (A.M. and N.A.) were responsible for data extraction, independently, with the final referee (MR.A.) intervening in the event of a disagreement. The information taken from each article included the authors name, date of publication, country of investigation, age of those involved, sample size of participants, sex, visfatin serum/plasma level, body mass index (BMI), CRP level, IL-6 level, TNF-α level, and forced expiratory volume in 1 s (FEV1). All parameters values were reported with mean and standard deviation.

### 2.4 Study quality assessment

The Newcastle-Ottawa Scale (NOS) was used to assess risk of bias for cohort studies. The NOS includes criteria such as selection, comparison, and outcome or exposure depending on the type of study (cohort or case–control series) (18). A star system is used, ranging from zero to nine stars. Thresholds were set based on the overall score, with 7 to 9 stars considered “low risk of bias,” 4 to 6 stars “unclear risk of bias,” and 3 stars or less “high risk of bias” (19).

### 2.5 Statistical analysis

The results were expressed in the form of mean ± SD. In order to determine the standard mean difference (SMD), transform the reported results to SD by converting them into a confidence interval and standard error (SE). The results were analyzed utilizing Comprehensive Meta-Analysis (CMA) software version 2 and the random-effects model, with *p* < 0.05 defining statistical significance, and MedCalc was used for meta-regression. The pooled SMD of BMI, age, FEV1, and IL-6 were calculated and the correlation of the above values with pooled SMD visfatin were reported using meta-regression analysis.

The Q-test and the I^2^ index were employed to assess heterogeneity, with a significant level of heterogeneity set at *p* < 0.10 (I^2^ < 25%, no heterogeneity; I^2^ between 25 and 50%, moderate heterogeneity; I^2^ between 50 and 75%, large heterogeneity; and I^2^ > 75%, extreme heterogeneity). An examination of sensitivity was undertaken to measure the effect of each study on the pooled effect size. The funnel plot examination and Egger’s regression test were utilized to analyze publication bias.

## 3 Result

### 3.1 Search results

**Figure 1:**
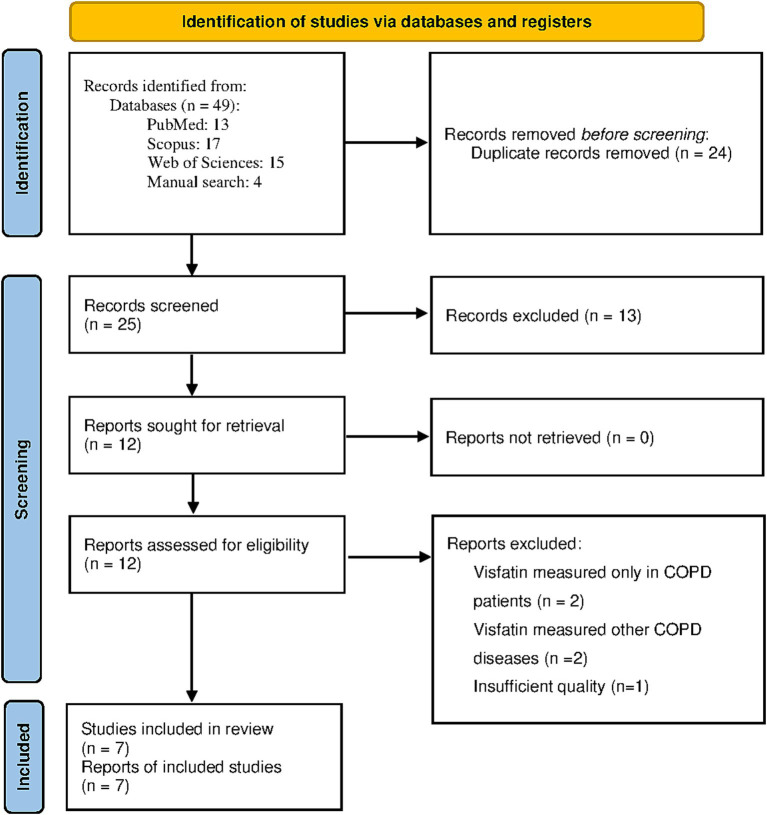
PRIMSA flow diagram.

The flowchart in Figure 1 displays the screening process of the current study. Initially, a total of 49 studies were identified. Twenty four studies were excluded as they were either duplicated or irrelevant. After examining the entirety of 25 studies, 13 were removed for not adhering to the criteria for inclusion. In addition, we removed four articles as the level of visfatin was measured only in COPD patients or other than COPD respiratory disorder (20–23). Also, one study was not included in the study due to insufficient quality (24). Finally, meta-analysis was performed with 7 studies (14, 25–30).

The case–control studies which were retrieved were published from 2009 up to 2022, with their aspects detailed in Table 2. Investigations were carried out in Turkey, Iran, Finland, China, and Mexico. The mean age of those identified with COPD was 63.59, compared to 61.71 in those without COPD. All research indicated a considerable variance in visfatin circulating concentration between COPD patients and control subjects, with 4 study results indicating a notable decrease for COPD patients and 2 study results a prominent increase (Table 2).

**Table 2:** Characteristics of included studies investigating the visfatin circulating level in COPD patients.

First Author, year	Country	Diagnosis	NOS (Stars)	Outcomes			
				Variable	Control	COPD	Intergroup differences (control vs. COPD)
Liu, 2009	China	ATS	7	Age (year)	70 ± 7	70 ± 7	NS
				Sex (M/F), n	(28/0)	(35/0)	-
				BMI (kg/m2)	22.3 ± 3.5	18.4 ± 2.3	<0.001
				FEV1	75.1 ± 8	59.5 ± 14.9	<0.001
				TNF-α (pg/ml)	9.44 ± 5.94	24.35 ± 9.99	<0.01
				CRP (ng/ml)	4.51 ± 3.01	17.46 ± 5.76	<0.01
				Visfatin (ng/ml)	1.88 ± 0.15	2.07 ± 0.18	<0.01
Eker, 2010	Turkey	GOLD	7	Age	63.84 ± 6.2	64.47 ± 7.9	NS
				Sex (M/F), n	(22/3)	(52/3)	-
				BMI (kg/m2)	24.80 ± 2.01	24.034 ± 4.2	NS
				FEV1	101.84 ± 15.51	49.61 ± 18.93	<0.001
				Alb (mg/dl)	4.51 ± 0.45	4.26 ± 0.53	<0.05
				TNF-α (pg/ml)	17.35 ± 3.7	21.87 ± 5.41	<0.001
				Leptin (ng/ml)	27.76 ± 14.32	6.27 ± 9.6	<0.001
				Visfatin (ng/ml)	3.27 ± 2.11	1.70 ± 1.47	<0.01
				FBG (mg/dl)	87.94 ± 8.21	91.74 ± 8.79	NS
				Insulin (μU/ml)	1.83 ± 1.17	3.71 ± 3.63	<0.05
				HOMA-IR	1.83 ± 1.17	3.71 ± 3.63	<0.01
				6MWT (m)	554.86 ± 38.85	371.03 ± 101.76	<0.001
				Smoking (pack/year)	0	51.54 ± 31	<0.01
Leivo-korpela, 2014	Finland	GOLD	7	Age (year)	-	59.5 ± 7.8	NS
				Sex (M/F), *n*	(41/0)	(43/0)	-
				BMI (kg/m^2^)	26.7 ± 3.9	25.8 ± 4.2	NS
				FEV1 (%)	-	53 ± 14	-
				Nesfatin(pg/ml)	44.91 ± 15.86	73.00 ± 17.60	NS
				IL-6 (pg/ml)	1.5 ± 1.2	1.5 ± 1.3	NS
				Visfatin (ng/ml)	8.9 ± 2.3	7.5 ± 1.5	<0.01
				MMP-9 (ng/ml)	33.9 ± 12.6	40.6 ± 17.3	<0.05
Perez-Bautista, 2018	Mexico	GOLD	7	Age (year)	67.26 ± 7.32	66.2 ± 7.79	<0.01
				Sex (M/F), *n*	(0/70)	(0/35)	-
				BMI (kg/m^2^)	26 ± 6	26.52 ± 6.13	NS
				FEV1 (%)	96.91 ± 13.36	52.40 ± 19.92	<0.001
				C-peptide (ng/ml)	0.107 ± 0.025	0.611 ± 0.126	<0.001
				Ghrelin (ng/ml)	0.063 ± 0.115	0.069 ± 0.021	<0.001
				GIP (ng/ml)	0.055 ± 0.012	0.095 ± 0.028	<0.05
				GLP-1 (ng/ml)	0.026 ± 0.003	0.063 ± 0.01	<0.001
				Glucagon (ng/ml)	0.186 ± 0.038	0.099 ± 0.02	<0.001
				Insulin (ng/ml)	0.166 ± 0.046	0.241 ± 0.074	NS
				Leptin (ng/ml)	0.705 ± 0.183	1.768 ± 0.646	<0.01
				PAI-1 (ng/ml)	15.049 ± 2.046	9.948 ± 3.077	<0.05
				Resistin (ng/ml)	0.348 ± 0.077	0.247 ± 0.137	<0.05
				Visfatin (ng/ml)	0.637 ± 0.106	0.258 ± 0.070	<0.001
				BODE	-	3.45 ± 2.86	-
				6MWT (m)	-	286.47 ± 174.23	-
				SaO2 (%)	-	89.54 ± 4.19	-
				Glucose	95.0 ± 10.0	101.86 ± 24.5	NS
				CRP	0.28 ± 0.32	0.56 ± 0.62	<0.01
Goktepe, 2020	Turkey	GOLD	7	Age (year)	43.04 ± 13.72	64.64 ± 8.58	<0.001
				^*^Sex (M/F), *n*	(30)	(30)	-
				BMI (kg/m^2^)	27.55 ± 4.16	25.61 ± 6.16	NS
				Resistin (ng/ml)	1.09 ± 0.56	1.89 ± 1.17	<0.05
				Chemerin, (ng/ml)	3.29 ± 2.49	4.68 ± 5.74	NS
				Visfatin (ng/ml)	8.03 ± 6.64	4.66 ± 6.22	<0.01
				Smoking (pack/year)	13.71 ± 5.39	54.16 ± 28.18	<0.001
Combay, 2021	Turkey	Clinical evaluation	7	Age (year)	60.05 ± 5.71	61.59 ± 5.91	NS
				Sex (M/F), *n*	(20/0)	(37/0)	-
				BMI (kg/m^2^)	23.67 ± 1.98	21.16 ± 2.84	<0.001
				FEV1 (%)	87.95 ± 4.55	31.05 ± 11.08	<0.001
				IL-6 (pg/ml)	1.23 ± 0.42	1.11 ± 0.08	<0.05
				MDA (nmol/mL)	0.64 ± 0.22	1.61 ± 0.24	<0.001
				Adiponectin (ng/mL)	22.51 ± 10.69	151.74 ± 93.68	<0.001
				Visfatin (ng/mL)	9.71 ± 1.36	8.37 ± 1.26	<0.001
Ghobadi, 2022	Iran	GOLD	8	Age (year)	56.27 ± 8.12	58.83 ± 9.47	NS
				Sex (M/F), *n*	(30/0)	(30/0)	-
				BMI (kg/m^2^)	26.90 ± 3.91	26.06 ± 5.30	NS
				FEV1 (%)	89.83 ± 8.38	53.13 ± 23.12	<0.001
				IL-6 (pg/ml)	55.05 ± 3.92	57.74 ± 2.21	<0.05
				Sirtuin-1 (ng/ml)	4.36 ± 0.74	3.5 ± 0.25	<0.05
				Visfatin (ng/ml)	2.881 ± 0.25	3 ± 0.49	<0.05
				Adjusted Visfatin (ng/ml)	2.54 ± 0.75	3.8 ± 0.63	<0.001
				Smoking (pack/year)	12.871 ± 13.43	29.13 ± 10.43	<0.001
				SpO2 (%)	-	92.83 ± 3.81	-
				CAT score	-	19.53 ± 9.20	-
				mMRC	-	1.97 ± 0.81	-

Alb, albumin; ATS, American Thoracic Society; BMI, body mass index; BODE, (Body Mass Index, Obstruction, Dyspnea, Exercise capacity); CAT, COPD Assessment Test; CRP, C-reactive protein; F, female; FBG, fasting blood glucose; FEV1, forced expiratory volume in 1 s; GIP, glucose-dependent insulinotropic polypeptide abbreviated; GLP-1, glucagon-like peptide-1; GOLD, Global Initiative for Chronic Obstructive Lung Disease; HOMA-IR, homeostatic model assessment for insulin resistance; IL-6, interleukin-6; M, male; MDA, malondialdehyde; MMP-9, matrix metalloproteinase-9; mMRC, Modified Medical Research Council; 6MWT, 6 min walking test; NOS, Newcastle–Ottawa quality assessment scale for case–control studies; PAI-1, plasminogen activator inhibitor-1; SpO2, O2 saturation; TNF-α, tumor necrosis factor alpha.

* Sex.

### 3.2 Quality assessment

The Newcastle-Ottawa scale was used to evaluate the quality of the studies. Evaluation was based on criteria such as the selection of the cohort, ensuring group comparability through design or analysis, the determination of exposure, and the assessment of outcomes of interest. The entry requirement for the study was a score of 6 or higher (Supplementary Table 2). The two researchers assessed the criteria for each paper in the study (A.M. and N.A.), independently. The results showed that one of the articles did not have the necessary quality to enter the analysis (24).

### 3.3 Study characteristics

Table 2 shows a summary of the characteristics of the included studies. In 7 included studies, 244 subjects were in the control group and 265 subjects were in the COPD group. All studies were in English and published between 2009 and 2022. The diagnosis of COPD was based on GOLD in 5 studies (14, 26–28, 30), ATS in one study (29), and clinical evaluation in one study (25). In 4 studies only male gender (14, 25, 27, 29), in one study only female gender (30), and in two studies both genders were selected (26, 28). Age and BMI values were reported in all studies. In one study, the FEV1 (%) value was reported only in the COPD group (14), while in 5 studies, it was reported in both the COPD and control groups (25–27, 29, 30). TNF-α values were reported in two studies (26, 29), IL-6 in three studies (14, 27, 28), CRP in two studies (29, 30), fasting blood glucose (FBG) in two studies (26, 30), and insulin in two studies (30). Visfatin values based on the severity of COPD disease (GOLD grade) were reported in 3 studies (26–28), in one study, the number of samples was mentioned in GOLD grade IV, and the other GOLD grades did not have sample size (28). In 5 studies, the participants were under 65 years old (14, 25–28) and in two studies, they were over 65 years old (29, 30). In 4 studies, subjects had a BMI over than 25 kg/m^2^ (14, 27, 28, 30) and in 3 studies under 25 kg/m^2^ (25, 26, 29). In 4 studies, FEV1 (%) value was over than 50 (14, 27, 29, 30) and in 2 studies it was under 50 (25, 26) (Table 2).

### 3.4 Effectiveness

#### 3.4.1 Visfatin circulating change level between COPD patients and control subjects

**Figure 2:**
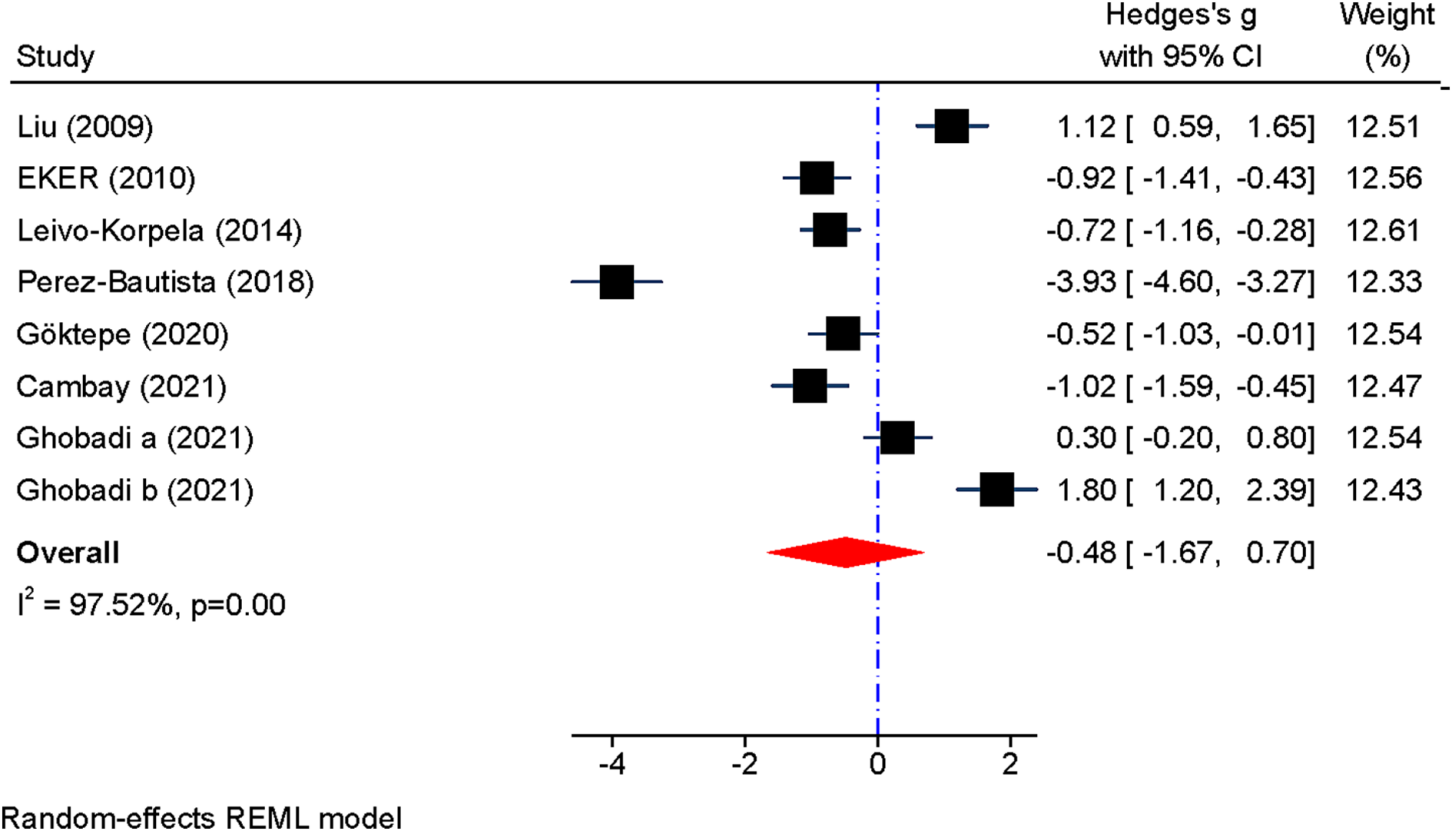
Forest plot showing the summary effect size for visfatin levels between COPD patients and control group.

Figure 2 displays the forest plot of visfatin concentration. Given the heterogeneity between studies, random effects models were used to analyze the results (I^2^ = 97%, *p* < 0.001). The pooled results showed that although the circulating concentration of visfatin was lower in patients with COPD, no significant difference was observed (SMD: −0.48 mg/L; 95% CI: −1.67 to 0.70; *p* = 0.43).

Subgroup analysis revealed that visfatin levels were significantly reduced in FEV1 less than 50% (SMD: −0.97, 95% CI: −1.35 to −0.60, *p* = 0.000); I^2^: 0%, *p* = 0.78). In addition, a significant decrease in visfatin levels was observed in GOLD grade I-II [(SMD: −0.43, 95% CI: −0.81 to-0.06, *p* = 0.022); I^2^ = 0%, *p* = 0.69]. However, subgroup analysis for BMI above and below 25 kg/m^2^ as well as age above and below 65 year had no effect on heterogeneity (Table 3).

**Table 3:** Subgroup analysis assessing the visfatin in COPD patient.

Variable Sub-grouped by		No. of arms	Effect size (SMD)	95% CI	*I*^2^ (%)	*p* for heterogeneity
Age	≥65	2	−1.41	−6.41, 3.59	99	0.000
	≤65	6	−0.19	−0.98, 0.60	92	0.00
BMI	≥25	5	−0.61	−2.17, 0.95	97	0.000
	≤25	3	−0.28	−1.66, 1.11	95	0.000
FEV1 (%)	≥50	4	−0.17	−2.43, 2.09	98	0.000
	≤50	2	−0.97	−1.35, −0.60	0	0.782
GOLD grade	Grade I-II	3	−0.43	−0.81, −0.06	0	0.692
	Grade III-IV	5	−0.13	−1.16, 0.89	90	0.000

BMI, body mass index; FEV1, forced expiratory volume in 1 *s*.

#### 3.4.2 Sensitivity analysis

**Figure 3:**
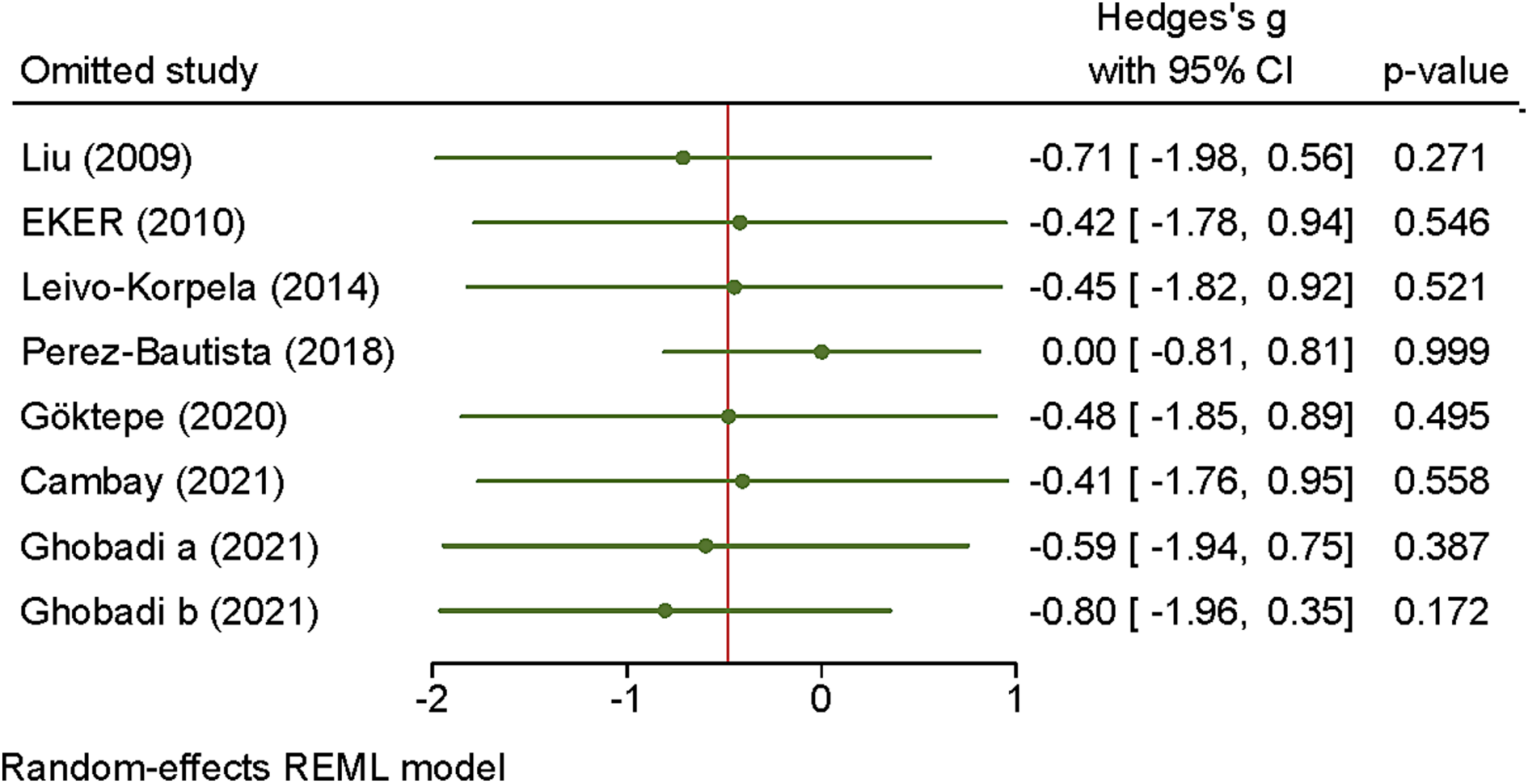
Sensitivity analysis of the association between visfatin and COPD. The influence of individual studies on the overall standardized mean difference (SMD) is shown. The middle vertical axis indicates the overall SMD. The circles represent the pooled SMD when the remaining study is omitted from the meta-analysis.

The sensitivity analysis further demonstrated that omitting of studies had no impact on effect size of visfatin (Figure 3).

#### 3.4.3 Publication bias

**Figure 4:**
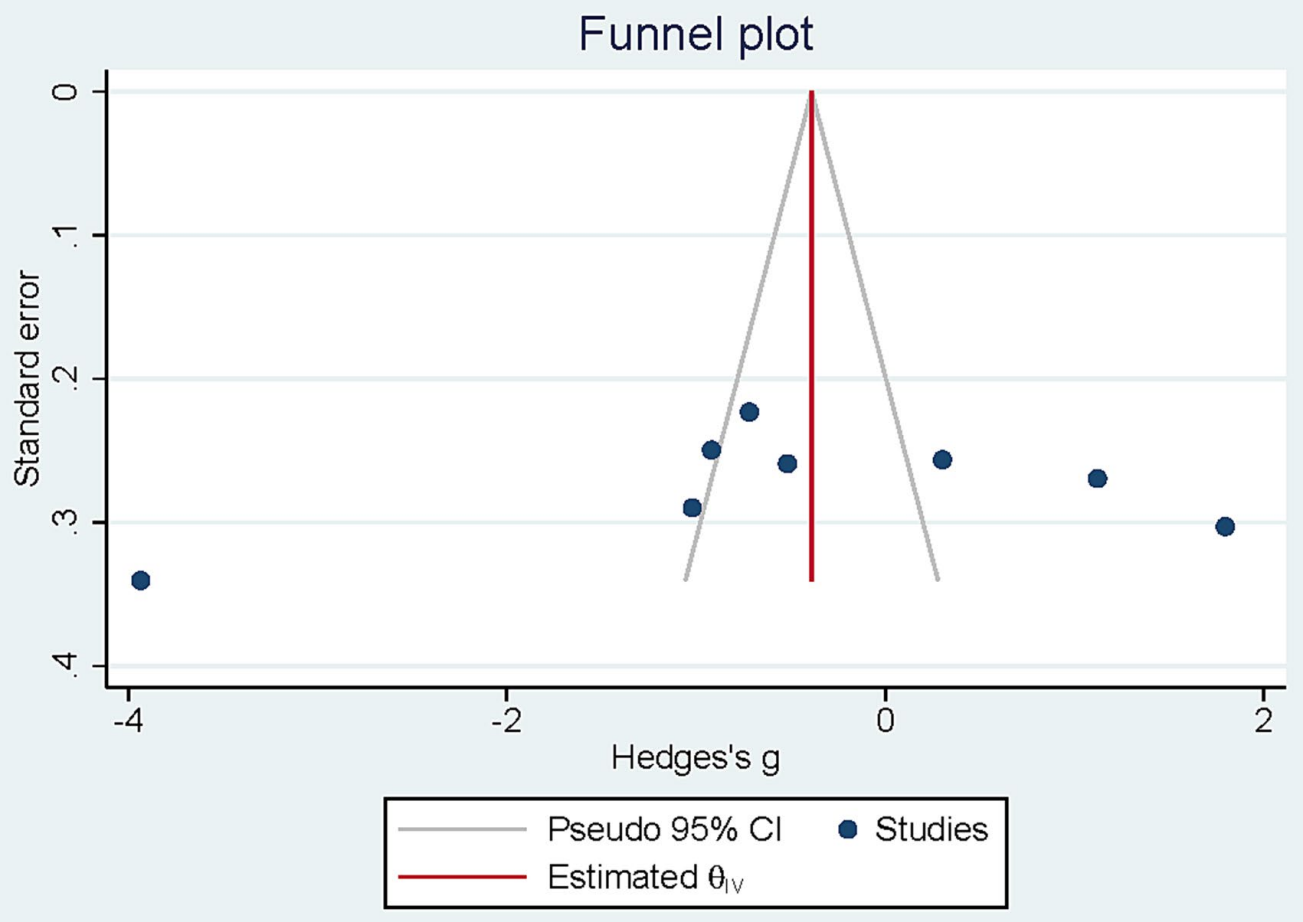
Funnel plot analysis.

The visual examination of the funnel plots demonstrate any asymmetry. Results from Egger test (*p* = 0.308) revealed no considerable publication bias (Figure 4).

#### 3.4.4 Meta-analysis of correlations between visfatin levels and inflammatory parameters

**Figure 5:**
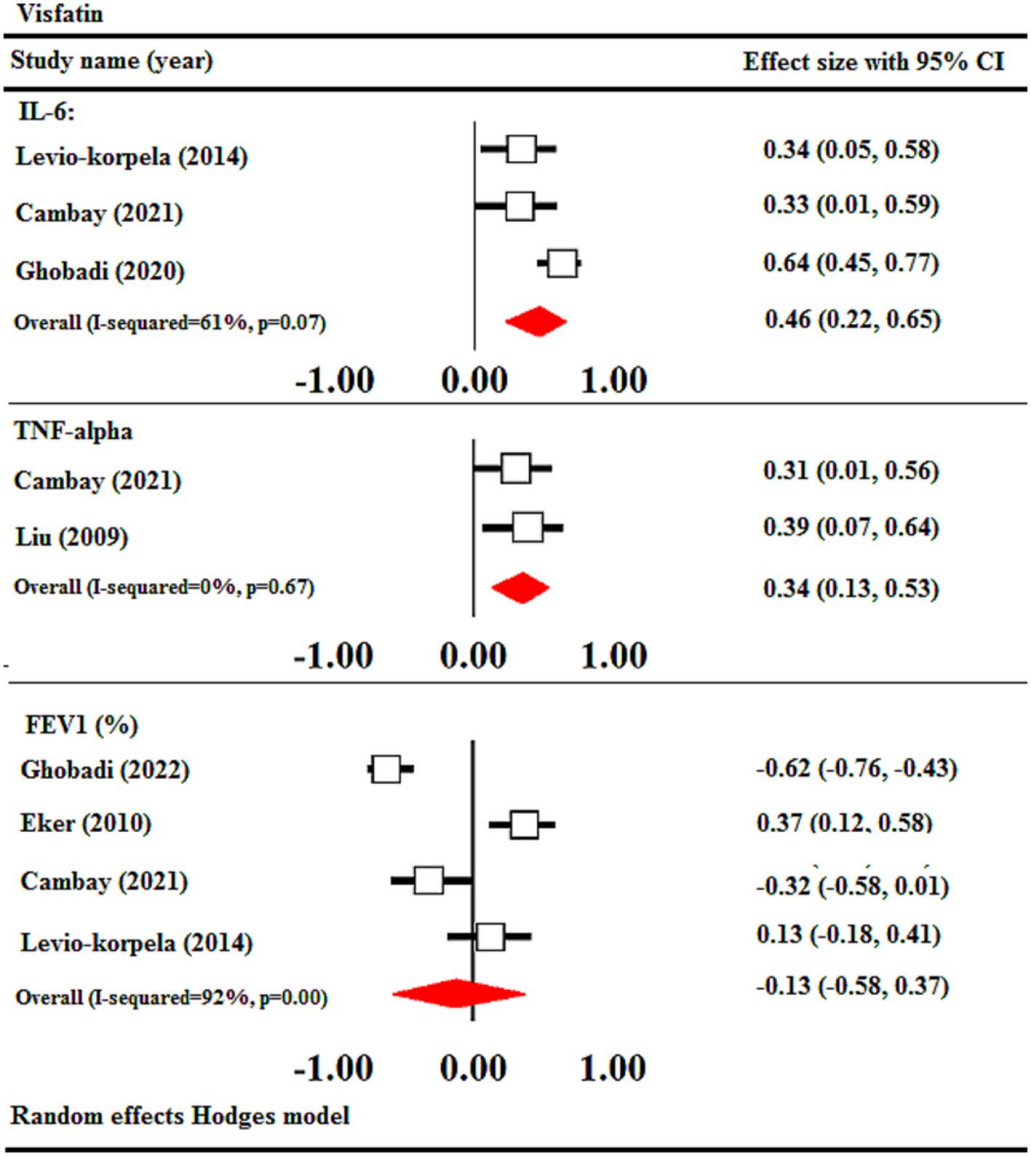
Meta-analysis of correlation coefficient between serum visfatin levels of COPD, IL-6, TNF-α, and FEV1. CI, confidence interval; IL-6, interleukin-6; TNF-α, tumor necrosis factor alpha; FEV1: forced expiratory volume in 1 s.

This study utilized correlational meta-analysis to examine the association between visfatin levels and inflammatory parameters. The findings of the meta-analysis demonstrated a notable positive association between visfatin concentrations and IL-6 (r = 0.46, 95% CI = 0.22 to 0.65, *p* < 0.001) and TNF-α (r = 0.34, 95% CI = 0.13 to 0.53, *p* < 0.01). No significant relationship was observed between visfatin levels and FEV1 (r = −0.13, 95% CI = −0.58 to 0.37) (Figure 5).

#### 3.4.5 Meta-regression

**Figure 6:**
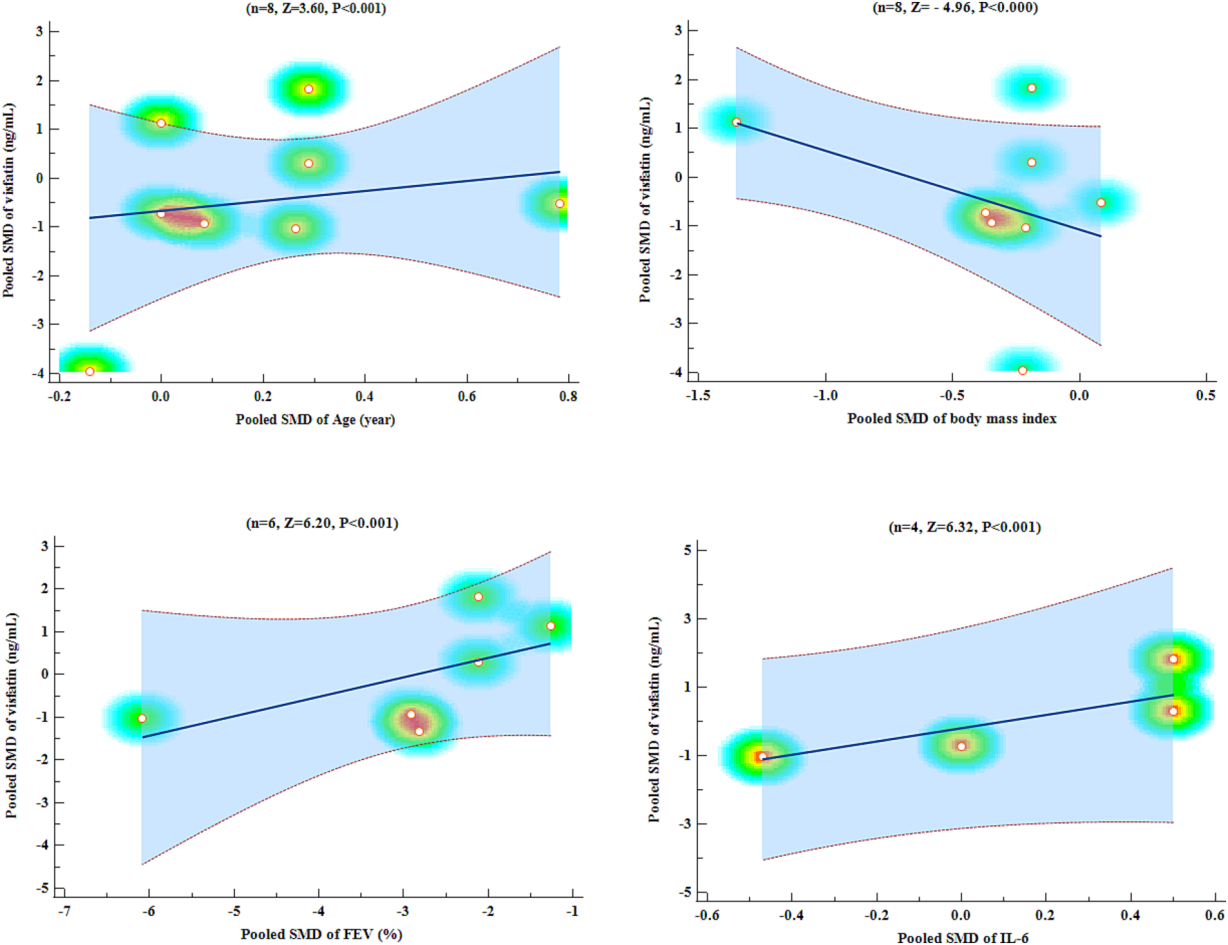
Meta-regression scatter plots.

An analysis of meta-regression was carried out to compare the pooled SMD of visfatin with pooled SMD of variables such as age, BMI, FEV1, and IL-6. Meta-regression analysis showed that pooled SMD visfatin was significantly associated with pooled SMD of age (Z = 3.60, *p* < 0.001), BMI (Z = −4.96, *p* < 0.001), FEV1 (Z = 6.20, *p* < 0.001), and IL-6 (Z = 6.32, *p* < 0.001) (Figure 6).

## 4 Discussion

The aim of this meta-analysis was to explore the potential association of circulating visfatin levels and COPD. From 7 relevant case–control studies, the following results were obtained: (1) visfatin level in COPD patients compared to healthy subjects did not show any significant difference based on meta-analysis, (2) correlation meta-analysis results indicated that there was a significant positive correlation between visfatin levels and IL-6 and TNF-α, and (3) results of the meta-regression analysis indicated that there was a direct correlation between pooled SMD visfatin and pooled SMD in age, BMI, FEV1, and IL-6 levels.

It has been noted through research that adipokines are agents related to chronic low-grade inflammation in metabolic disorders and inflammatory diseases (31). Numerous studies on humans and animals have revealed modified adipokine levels in inflammatory lung diseases such as asthma and COPD (31). Moreover, in critically ill patients, elevated adipokine levels are typically linked to organ malfunction and tissue inflammation (32). It has been established that visfatin plays a role in certain chronic inflammatory diseases (14), however, its association with COPD is not clear. As with those with obesity and insulin resistance (33, 34), COPD patients show both elevated and reduced visfatin levels when compared to healthy individuals (25, 27).

Despite the findings of the recent meta-analysis showing no significant difference in visfatin levels between COPD patients and the control group, it is possible that the heterogeneity among the studies impacted the outcome. The findings of the subgroup analysis indicated that as FEV1 levels decreased and disease severity reached GOLD grade I-II, the heterogeneity within the study decreased. The findings demonstrated that different variables, like the diseases severity, influence visfatin outcomes in COPD patients and must be taken into account during patient selection. Visfatin is predominantly expressed in visceral adipose tissue. Results on the relationship between visfatin level and BMI are conflicting, as obese people have been observed to have higher, lower, or unchanged visfatin levels. It has been documented in some studies that underweight COPD patients had higher visfatin levels (35, 36). The cause of elevated visfatin levels in COPD patients with reduced BMI is not fully understood, yet it is speculated that severe systemic inflammation is likely to be a significant contributor (37).

A correlation meta-analysis demonstrated a significant positive association between visfatin levels and inflammatory factors such as IL-6 and TNF-α. Furthermore, meta-regression results indicated a significant correlation between visfatin level and IL-6 levels. While visfatin expression is mainly derived from visceral adipose tissue, macrophages, and dendritic cells are also able to produce visfatin (38). On the other hand, among the cells that play a key role in the pathophysiology of COPD are neutrophils and macrophages (7). Macrophages are considered as the main source of cytokines such as IL-6 and TNF-α. It is believed that macrophage activation causes the release of adipokines in patients with COPD (36). In addition, it has been observed that inflammatory cytokines such as IL-6, TNF-α, and IL-1β can stimulate visfatin expression (39). Interestingly, it has been revealed that visfatin itself induces the production of inflammatory factors (IL-6 and TNF-α) (36).

Overall, it appears that visfatin is pro-inflammatory factor in chronic diseases. An ongoing debate exists regarding visfatin’s pro-inflammatory nature and its decreased concentrations in COPD patients. This controversy could be explained by a number of factors. First, high and low levels of adipokines in chronic inflammatory diseases have been acknowledged to have an impact on the immune system (40). Lower concentrations of some adipokines have been linked to recurrent lung infections due to compromised immune function (14). Using GOLD criteria to evaluate visfatin levels in COPD patients could have been more precise as a study found heightened visfatin levels in severe GOLD grade (27). Finally, the meta-analysis investigated visfatin levels in serum/plasma samples of COPD patients. Previous research has demonstrated that adipokines can produce systemic and local effects. Investigating visfatin levels in the lung tissue appears to provide more precise assessments of alterations in its levels.

## 5 Limitations

This study had some limitations. At first, there were a limited number of studies included in the meta-analysis, therefore more studies are required for an in-depth analysis. Because only articles published in English were selected for the study, the small number of articles included may have an impact, which could be considered a limitation of the study. Second, there might be bias in the current results because some studies were not included or some data was inaccessible. The connection between visfatin levels and disease severity in COPD patients was particularly evident in this case. Within the meta-analysis, research on visfatin outcomes were analyzed with reference to GOLD grade in certain studies. Moreover, the studies failed to address whether COPD patients were hospitalized during their stable or exacerbation phases, possibly impacting the integrity of the results. The study was limited by the absence of details on patient’s treatment conditions, such as their current medication, duration of illness, and etc. Previous research has demonstrated that visfatin plays a pivotal role as a pro-inflammatory factor. Therapeutic interventions in patients with COPD may have been effective in modulating the results of visfatin levels, which should be considered as an influential factor. Additionally, changes in visfatin levels may be influenced by both comorbidities and metabolic conditions within COPD patients, variables that were not taken into consideration in the included studies.

## 6 Conclusion

The pooled analysis of visfatin levels in COPD patients versus healthy subjects indicated no notable variance between the groups. The high levels of heterogeneity among the studies may have played a role in this lack of differentiation. The subgroup analysis conducted using FEV1 and disease severity (GOLD grade) revealed them as primary sources of heterogeneity in the studies, while acknowledging the potential influence of other variables like BMI and age. It is suggested that additional studies be conducted in order to better interpret the results and identify the sources of heterogeneity. In the present study, a correlation meta-analysis indicated a positive association between visfatin levels and inflammatory factors including IL-6 and TNF-α. In future research studies, it may be beneficial to closely analyze visfatin levels in patients with COPD while taking into account their inflammatory conditions.

## Data Availability

The original contributions presented in the study are included in the article/Supplementary material, further inquiries can be directed to the corresponding author.
