# Supplementary material for: Circulating visfatin concentrations in patients with chronic obstructive pulmonary disease: systematic review and meta-analysis
Source: Front Med (Lausanne). 2025 Jan 7;11:1432025. doi: 10.3389/fmed.2024.1432025 (PMC11752917; doi:10.3389/fmed.2024.1432025)
Supplement: Supplementary file 1 [file Data_Sheet_1.docx]

Supplementary data:

| **Supplementary Table 1.** Search strategy in different databases. | | |
| --- | --- | --- |
| Databases | Search strategies | Number |
| PubMed | ("Nicotinamide Phosphoribosyltransferase"[Mesh] OR “Visfatin”[tw] OR “Nicotinamide Phosphoribosyltransferase”[tw] OR “NAMPT Protein”[tw] OR “Pre-B-Cell Colony-Enhancing Factor”[tw] OR (Colony-Enhancing Factor AND Pre-B-Cell) OR “Pre B Cell Colony Enhancing Factor”[tw] OR “PBEF”[tw] OR “NAmPRTase”[tw] OR “NMN Pyrophosphorylase”[tw])  AND  ("Pulmonary Disease, Chronic Obstructive"[Mesh] OR “Chronic Obstructive Pulmonary Diseases”[tw] OR “COPD”[tw] OR “COAD”[tw] OR “Chronic Obstructive Airway Disease”[tw] OR “Chronic Obstructive Pulmonary Disease”[tw] OR (Airflow Obstruction AND Chronic) OR (Airflow Obstructions AND Chronic) OR “Chronic Airflow Obstructions”[tw] OR “Chronic Airflow Obstruction”[tw] OR “emphysema”[tw] OR “chronic bronchitis”[tw]) | 13 |
| Scopus | ( TITLE-ABS-KEY ( "Visfatin" OR "Nicotinamide Phosphoribosyltransferase" OR "NAMPT Protein" OR "Pre-B-Cell Colony-Enhancing Factor" OR ( colony-enhancing AND factor AND pre-b-cell ) OR "Pre B Cell Colony Enhancing Factor" OR "PBEF" OR "NAmPRTase" OR "NMN Pyrophosphorylase" )  AND  TITLE-ABS-KEY ( "Chronic Obstructive Pulmonary Diseases" OR "COPD" OR "COAD" OR "Chronic Obstructive Airway Disease" OR "Chronic Obstructive Pulmonary Disease" OR ( airflow AND obstruction AND chronic ) OR ( airflow AND obstructions AND chronic ) OR "Chronic Airflow Obstructions" OR "Chronic Airflow Obstruction" OR "emphysema" OR "chronic bronchitis" ) ) | 17 |
| Web of sciences | (TITLE-ABS-KEY( "Visfatin" OR "Nicotinamide Phosphoribosyltransferase" OR "NAMPT Protein" OR "Pre-B-Cell Colony-Enhancing Factor" OR (colony-enhancing AND factor AND pre-b-cell) OR "Pre B Cell Colony Enhancing Factor" OR "PBEF" OR "NAmPRTase" OR "NMN Pyrophosphorylase")  AND  TITLE-ABS-KEY("Chronic Obstructive Pulmonary Diseases" OR "COPD" OR "COAD"  OR "Chronic Obstructive Airway Disease" OR "Chronic Obstructive Pulmonary Disease" OR (airflow AND obstruction AND chronic) OR (airflow AND obstructions AND chronic) OR "Chronic Airflow Obstructions" OR "Chronic Airflow Obstruction" OR "emphysema" OR "chronic bronchitis") | 15 |

| **Supplementary Table 2.** The Newcastle-Ottawa scale for case – control studies | | | | | | | | | |
| --- | --- | --- | --- | --- | --- | --- | --- | --- | --- |
| Study name (year) | case definition | Representativeness | Selection of Controls | Definition of Controls | Comparability | Ascertainment of exposure | Same method of ascertainment | Non-Response rate | Overall |
| Liu (2009) | 1 | 1 | 0 | 1 | 1 | 1 | 1 | 1 | 7 |
| Eker (2010) | 1 | 1 | 0 | 0 | 1 | 2 | 1 | 1 | 7 |
| Leivo-Korpela (2014) | 1 | 1 | 0 | 1 | 1 | 1 | 1 | 1 | 7 |
| Pérez-Bautista (2018) | 1 | 1 | 0 | 1 | 1 | 1 | 1 | 1 | 7 |
| Gotepeke (2020) | 1 | 1 | 0 | 1 | 1 | 1 | 1 | 1 | 7 |
| Salman (2020) | 0 | 0 | 0 | 0 | 1 | 1 | 1 | 1 | 4 |
| Cambay (2021) | 1 | 1 | 0 | 0 | 1 | 2 | 1 | 1 | 7 |
| Ghobadi (2022) | 1 | 1 | 1 | 1 | 1 | 1 | 1 | 1 | 8 |
